# The Ugandan youth public health ambassador program: a community-based public health initiative

**DOI:** 10.3389/fpubh.2025.1640598

**Published:** 2025-12-03

**Authors:** Heather Wipfli, Abigail Kim, Cecilia Alonyo, Kyra Guy, Andre A. Fabian, Samantha K. Gillis, Ray Wipfli, Penninah Tumuhimbise, Kenneth Odur

**Affiliations:** 1 USC Global Research, Implementation, and Training Lab, University of Southern California, Los Angeles, CA, United States; 2 Energy in Action, Kampala, Uganda; 3 Children’s Chance International, Central Division, Lira Municipal Council, Lira, Uganda

**Keywords:** public health, sub-Saharan Africa, community-based, adolescent, education

## Abstract

**Background:**

In 2020, the Global Research, Implementation, and Training (GRIT) Lab at the University of Southern California was forced to quickly shift its large community-based youth public health training programs to respond to the COVID-19 realities. This resulted in the implementation of the Youth Public Health Ambassador (YPHA) program, initially launched in Lira district, Uganda, and since expanded to include Kibera, Nairobi, Kenya. The program serves as a model for other youth-focused, community-based public health education, research, and policy advocacy programming effective in both rural and urban settings.

**Program components:**

The YPHA Program consists of a multi-year training initiative led by public health students and professionals from East Africa and the United States. The program identifies and empowers youths (aged 18–24) as peer educators, data collectors, and agents of change. The program consists of three distinct components: basic public health education, implementation of a community-based participatory research study, and community outreach and data dissemination. Ambassador proficiency in public health and leadership skills were assessed throughout.

**Program outcomes:**

Upon program completion, YPHAs showed greater mastery and indicated having higher confidence in educating their community members in all topic areas. YPHAs were also able to successfully conduct a comprehensive community health assessment by collecting and analyzing data from households, health facilities, and schools. YPHAs then disseminate their results within their communities and to local and national policymakers, the Ministries of Health, and the United States Embassy.

**Conclusion:**

The program’s findings will hopefully encourage further investment in youth-centered health promotion programming, further incorporation of youth-led programs for low-income communities, and youth-driven policy advocacy.

## Background

People aged 30 and under make up 47% of the world population. In many low- and middle-income (LMICs) countries, this percentage rises to over 70% (1). Despite their demographic significance, adolescents and young adults, especially those living in LMICs, continue to experience profound barriers to health and economic stability, including limited access to quality education, health care, and economic opportunity. Nevertheless, young people remain markedly underrepresented in decision-making settings, including local, national, and international discussions related to health and sustainable development (2).

The importance of engaging youth in health and sustainable development research and programming is widely recognized. Studies show that young people possess deep insights into their communities’ needs and challenges, contribute innovative solutions to persistent public health problems, and effectively mobilize their peers to drive awareness and behavior change (3–5). Involving youth in public health decision-making enhances the cultural and educational relevance of policies and programs strengthens social networks, mobilizes community capacity, promotes sustainability, and increases the likelihood of its long-term success and policy influence (6–9).

Despite the demonstrated benefits, numerous barriers hinder meaningful youth engagement in decision-making, especially in sub-Saharan Africa. These include inequitable access to representation, limited digital access, the high cost of youth-focused programming, and persistent skepticism toward youth perspectives among senior leaders (2). These COVID-19 pandemic further exacerbated these challenges, leaving a substantial segment of society underrepresented in governance and resulting in policies that fail to address the needs of current and future generations (10, 11).

Community-based participatory research (CBPR) frameworks have proven effective for engaging marginalized and hard-to-reach populations, including youth, in public health decision-making (12, 13). CBPR involves equitable collaboration between researchers and community members throughout the research process, from developing questions to the interpretating, disseminating, and applying findings (14). This approach deepens understanding of health issues within their social, political, economic, and cultural contexts, improving community health and well-being (15). It also fosters local capacity building, knowledge exchange, and empowerment at both individual and community levels (16, 17). While CBPR methodologies have been well developed in Canada and the United States, there remains a pressing need to rigorously evaluate their effectiveness within sub-Saharan Africa, particularly among youth (18–20).

In response, the Youth Public Health Ambassador Program (YPHA), launched during the COVID-19 pandemic, employed peer-led CBPR strategies to address barriers to youth participation in public health initiatives in Lira district, Northern Uganda. This paper outlines the program’s objectives and components, describes its CBPR strategies, and presents ambassador evaluations and learning outcomes. It concludes with lessons learned and recommendations for empowering previously excluded youth in public health decision-making and programming. As demonstrated by the YPHA program, equipping marginalized youth with technical and leadership skills to influence policy can lead to meaningful improvements in their lives and communities.

## Program setting, partnership, design and implementation

### Program setting

Lira District, situated in the northern region of Uganda, was the epicenter of protracted conflict between the Lord’s Resistance Army (LRA) and the Ugandan government. At the height of the conflict, from 1996 to 2006, approximately 1.7 million people were displaced and resided in camps across the region (20). In the post conflict era, the district’s population remains predominantly engaged in subsistence farming, with an estimated 69% of households classified as severely food insecure. Notably, 78% of the population is under 30 years of age and roughly 80% of youth aged 15–24 years live in rural areas (21).

Youth in Lira face substantial barriers to health and economic stability. Approximately 78% of people over 15 years of age have not completed secondary education (senior 4), while nearly 15% of girls aged 10–17 are married and/or have given birth to at least one child (22, 23). The COVID-19 pandemic further exacerbated these vulnerabilities, with widespread school closures and the suspension of public health programming contributing to heighted risks of child marriage, unplanned pregnancy, domestic violence, substance abuse, and HIV (24).

### Program partners

The YPHA program is a partnership between the Global Research, Implementation, and Training (GRIT) Lab at the University of Southern California (USC), the youth-led international non-profit Energy In Action (EIA), and the Lira-based community organization Children’s Chance International (CCI). Since 2017, USC, EIA, and CCI-Uganda have jointly implemented adolescent and young adult public health promotion activities in Lira District. Between 2017 and 2019, the consortium organized annual weeklong, community-based youth soccer and public health camps, engaging over 1,000 local youth each year in peer-led public health educational activities.

In 2020, COVID-19 related restrictions necessitated a strategic shift in program implementation. Program partners transitioned from a model emphasizing broad engagement to one focused on sustained, intensive collaboration with a smaller cohort of young adults. These youth leaders, in turn, served as conduits for disseminating health information and promoting behavior change within their respective communities.

### Program design

The YPHA program was conceived as a longitudinal initiative to train and empower young leaders to actively participate in public health and sustainable development policy and programming in their communities. Building on the peer-led framework of the partners’ previous public health camps, the program emphasized community-based engagement rooted in the principles of social justice and equity and employed best practices related to respect, fairness, and cultural harmony (15, 18, 25, 26).

**Figure 1:**
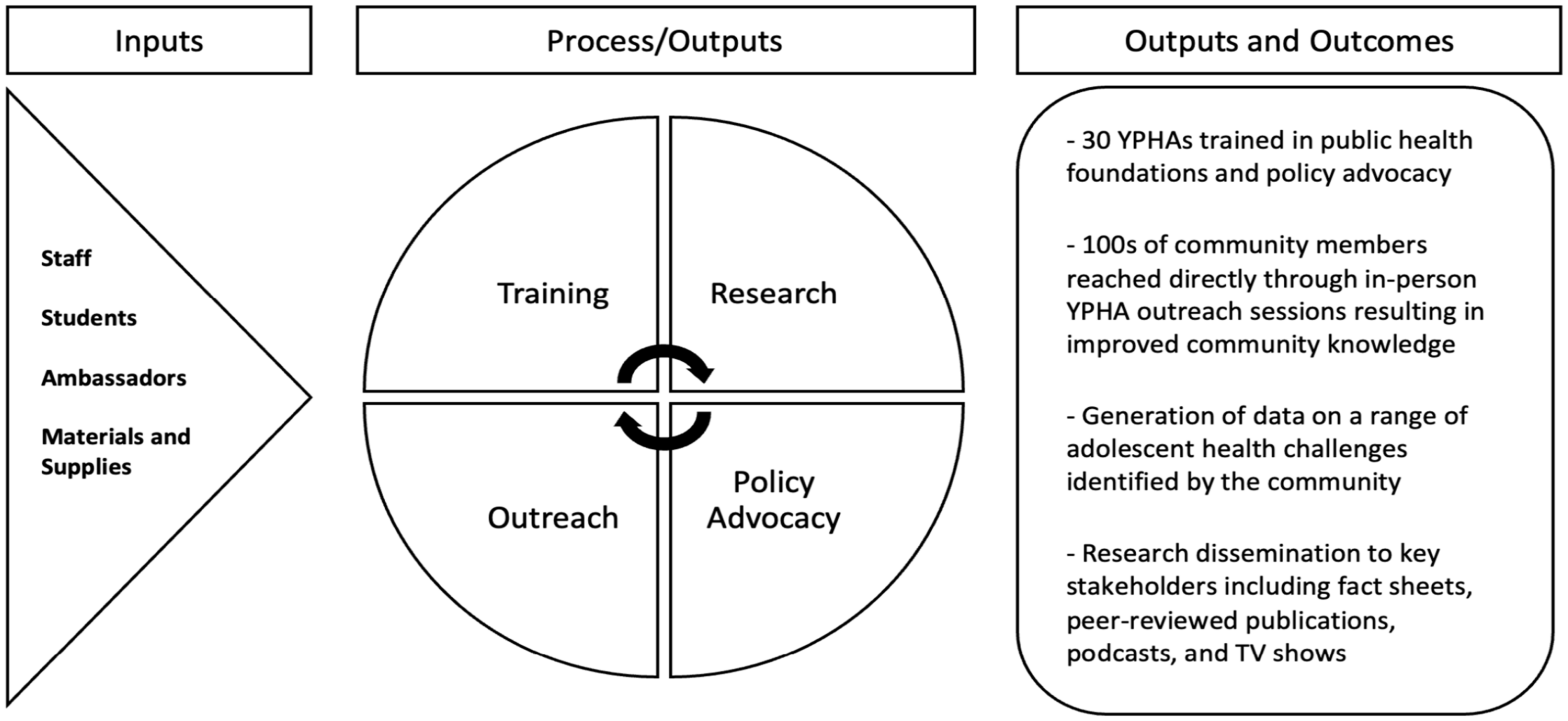
Logic model for the Uganda youth public health ambassadors program components and outputs.

**Figure 2:**
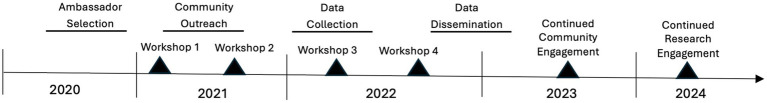
Program timeline from the Uganda youth public health ambassadors program.

The program was structured around three core components: (1) education in community-identified public health topics (e.g., sanitation, risky behaviors, sexual and reproductive health, and mental health); (2) training and practice in CBPR including data collection, analysis, and dissemination to advance community-identified health priorities; and, (3) coaching in public speaking, advocacy, and educational outreach to improve community-based public health practices (Figure 1). Although initially planned as an 18-month intervention designed to span the COVID-19 lock-down period, formal program activities were ultimately extended and continued over a 3 year period (Figure 2).

### Ambassador recruitment and demographics

Due to restrictions on public gatherings at the on set of the COVID-19 pandemic in 2020, the program launched with an initial cohort of 30 youth aged 17–23 years. Participants were recruited from 3 sub-counties (Agweng, Ayami, and Aromo) in Lira District, Uganda. To recruitment process was coordinated by community volunteers from CCI, who collaborated closely with local council representatives at both the sub-county and village levels to introduce the program, outline its objectives, and explain the selection process. CCI volunteers also held meetings with parents and prospective participants to share program information; interested youth were subsequently guided through the eligibility requirements and key expectations.

Eligibility criteria included being aged 17–22, a permanent resident of Lira District, literate in both English and Lango, available and willing to volunteer, and demonstrating a strong commitment to community service.

Selection and recruitment of youth was done through a rigorous yet transparent process led by local council chairpersons, parents, and CCI community volunteers. Assessment activities included interviews, problem solving exercises, and brief speeches presented before the selection panel. In alignment with the program’s emphasis on gender equity and community empowerment, female participants were intentionally prioritized (*n* = 22) over men (*n* = 7), resulting in a total of 29 trained ambassadors.

The selected ambassadors reflected a diverse range of educational backgrounds. At the time of recruitment, one ambassador had completed lower secondary school (O-levels), 19 were currently enrolled in secondary education, one remained in primary school, four had left attending school prior to the pandemic-related closures, and five were no longer pursing formal education (Table 1).

**Table 1:** Demographic characteristics of participants from the youth public health ambassador participants program in Uganda (*N* = 29).

Characteristic	*n*	%
Gender		
Female	20	68.9%
Male	9	31.03%
Sub-county		
Agweng	8	27.6%
Ayami	9	31.03%
Aromo	12	41.4%
Age group		
15–17	12	41.4%
18–23	17	58.6%
Education status		
Currently in school	20	68.9%
Not in school	9	31.1%
Education level completed		
Primary	2	6.9%
Secondary	17	58.6%

### Training workshops

Between January 2021 to July 2022, YPHAs participated in a series of multi-day, peer-led training workshops. The curriculum covered public health risks, community outreach and facilitation, research methods, data analysis, data presentation, public speaking, and leadership skills. Each workshop incorporated interactive, experiential learning techniques supported by visual aids to facilitate comprehension. Participants received an English-Lango workbook, developed collaboratively by GRIT Lab, CCI and EIA. Workbooks were tailored based on each workshop theme and illustrated with culturally relevant concept art and imagery.

Workshop pedagogy followed the “I Do, We Do, You Do” model, allowing participants to gradually increase autonomy until mastery of specific competencies was achieved (27). Practical skills included menstrual health education and reusable pads construction; sanitation and hygiene promotion through handwashing demonstrations and building portable handwashing stations; and malaria prevention, including net installation and case identification. To support knowledge retention between training workshops, EIA and GRIT Lab volunteers developed concise pamphlets addressing key topics including WASH and disease spread, mental health, nutrition, risky behavior, and reproductive health. The pamphlets were distributed to ambassadors by the CCI staff.

Beyond technical information, workshops also emphasized socialization and fun. Activities included ice-breakers, team-building exercises, dance sessions, songwriting, and social media challenges. Training workshops were facilitated by youth trainers, including EIA staff (many of whom are students at Makerere University) and USC GRIT student volunteers. While initial sessions required that the US-based student volunteers join via Zoom due to travel restrictions, subsequent workshops were attended in person, fostering cross-cultural exchange and peer networking. Ambassadors hosted visiting Kampala-based and international facilitators in their homes (grass hut compounds), providing opportunities for deeper community immersion, trust-building, and enhanced program visibility.

### Community-based participatory research

Integrating formal CBPR was a central goal of the YPHA program. To research component began with a Delphi exercise during the inaugural workshop, through which ambassadors identified the most pressing health challenges within their communities. This iterative prioritization process employed successive questionnaires to achieve consensus (28). The ambassadors identified key areas of concerns, including malaria, poor nutrition, limited access to sexual and reproductive health education and services, and inadequate mental health support.

In the second training workshop, participants were introduced to fundamental research design principles and tasked with developing a draft study protocol, including identifying a target population, a sampling strategy, and a data collection method. They were also trained in CBPR-related ethical considerations including informed consent, protection of privacy, and confidentiality. Following the workshop, YPHAs collaborated with GRIT Lab student volunteers to refine study materials, which were subsequently submitted for ethics review and approval to USC, Gulu University, and the Uganda National Council for Research and Technology (UNCST).

The first study was a cross-sectional survey examining malaria-relates knowledge, attitudes, and behaviors among 500 young mothers received malaria net through the YPHA World Malaria Day activities in 2021 and 2022. Adapted from validated instruments such as the Peace Corps’ standard malaria survey, the tool assessed accessed household access to malaria prevention resources, daily preventive practices, and attitudes toward prevention efforts (29). Local council leaders assisted in identifying participants from households at greatest risk of malaria morbidity and mortality, including pregnant women, children under 5, orphans, individuals with disabilities, and older adults persons (aged 60+). YPHAs, working alongside EIA and CCI staff, obtained written informed consent in Lango and administered the surveys during net distribution. The study results, later shared with community leaders and published in the international peer-reviewed literature, revealed widespread shortages in bed net availability and substantial behavioral and logistical barriers to consistent use (30).

The second study collected quantitative and qualitative data on nutrition, mental health, and access to sexual and reproductive health education and services across households, schools, and healthcare facilities in Lira District. Following ethics approval, ambassadors attended a workshop in January 2022 to review study procedures and practice data collection using digital tablets. Each ambassador received printed consent and data collection forms, as well as a bicycle to facilitate mobility during fieldwork.

Between February and April 2022, YPHAs collected data from 300 households, 306 adolescents, 19 schools, and 6 healthcare facilities across six parishes in Agweng, Ayami, and Aromo sub-counties. CCI staff conducted weekly check-ins to resolve challenges and coordinate data uploads to a secure cloud at the CCI office in Lira, after which the data was cleaned and analyzed by GRIT Lab student in Los Angeles. Data analysis workshops held in June 2022 enabled collaborative interpretation between YPHAs and GRIT lab students. Findings were subsequently disseminated at the local, regional, national, and international levels, including being published in peer-reviewed journals, with each ambassador listed as a co-author (31). In 2024, YPHAs independently initiative an additional CBPR project focusing on road safety.

### Community outreach and data dissemination

A key objective of the YPHA program was to empower young leaders to serve as educators, facilitators, and advocates for improved health and sustainable development. Knowledge translation and community action were integral components of the program from its inception. During the first workshop, ambassadors received ‘community outreach kits’, including shirts, badges, and materials needed to host reusable menstrual pad making and construction of handwashing stations workshops.

In the month following their initial training, YPHAs conducted several community workshops. As the program gained visibility, Peace Corps Uganda reached out and provided the YPHAs with malaria-related educational materials and demonstration nets. Between 2021 and 2023, Peace Corps Uganda donated more than 5,000 malaria nets for YPHA-led distribution during World Malaria Day events. Building on this success, YPHAs launched a soap-making social enterprise, teaching community members how to make and sell the liquid soap as both a hygiene intervention and an income-generating activity.

**Figure 3:**
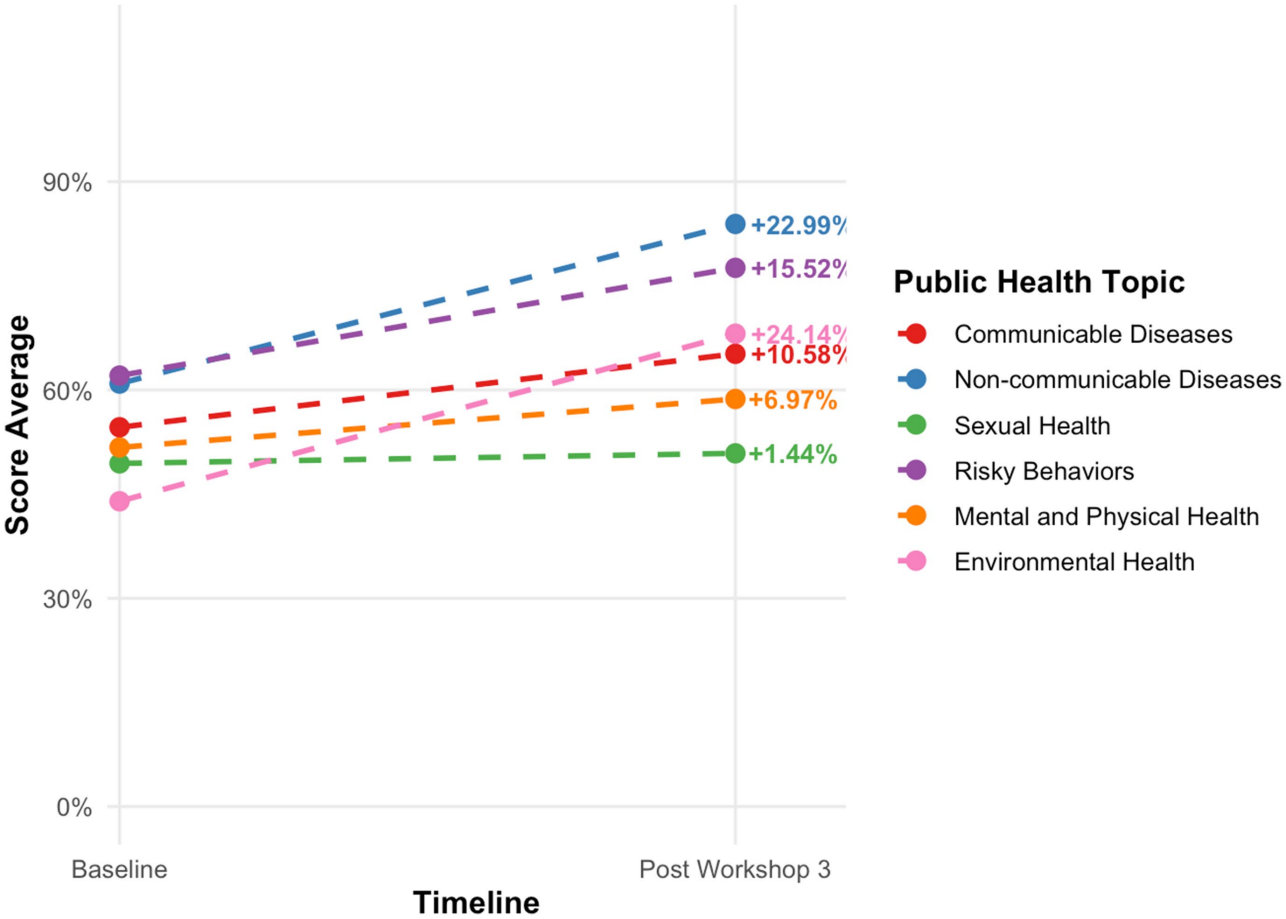
Changes in mean public health knowledge scores from baseline survey to post workshop three survey among youth public health ambassadors. Values represent mean composite scores for each domain. Paired sample *t*-tests were used to assess changes with a *p* < 0.05 considered statistically significant.

**Figure 4:**
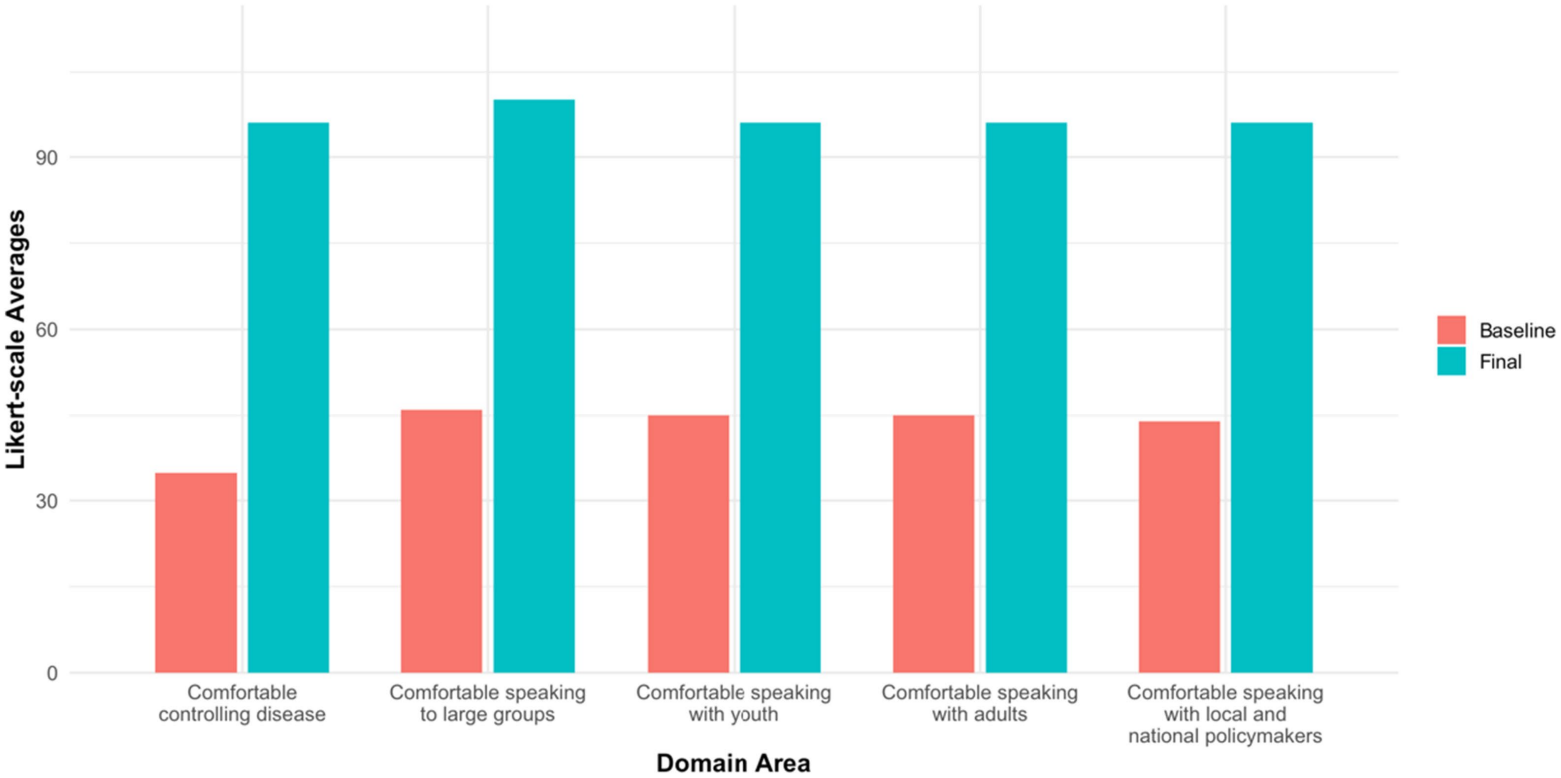
Changes in confidence levels from baseline survey to post workshop three survey among youth public health ambassadors. Values represent mean Likert-scale confidence ratings.

Ambassadors also played an active role in data dissemination and policy advocacy. The fourth workshop, held in June 2022, introduced stakeholder mapping and culminated in a district-wide press event where YPHAs presented research findings and policy implications to local community leaders, district officials, and the public. Five Ambassadors were invited by the National Malaria Commission to present at a workshop in Kampala, while another five presented findings from the adolescent health study to the Ugandan Parliamentary Committee for Health, the Ministry of Health, the Makerere University School of Public Health (MakSPH), and the US Embassy (Figures 3, 4). They also appeared on *Health Pot*, a nationally televised program supported by MakSPH (32). National media outlets provided additional coverage of community events.

## Program evaluation

A mixed-methods evaluation framework was used to assess the feasibility, acceptability, and educational impact of the YPHA program, integrating quantitative surveys and qualitative focus group discussions (FDGs).

Baseline and follow-up surveys were administered to all 29 ambassadors at the first training workshop in January 2021 and again after training workshop three. Surveys were conducted in English, with translation to Lango provided as needed. Data collection was led by EIA and CCI staff, with technical support from USC GRIT lab student volunteers.

Surveys were designed to measure changes in public health knowledge, confidence, and self-efficacy in abilities to disseminate information to others, and satisfaction following involvement in the program. Public health survey topics were organized into multiple choice, true/false, and select all that apply questions surrounding 11 topic domains (e.g., risky behaviors, communicable diseases, nutrition, mental health). Participant satisfaction questions followed a 5-point Likert scale ranging from (1-Very Dissatisfied to 5-Very Satisfied). Baseline surveys also collected demographic data from participants.

All paper-based responses were scanned and entered into Qualtrics by GRIT and EIA volunteers. Data cleaning and analysis were conducted using Microsoft Excel (2021) and IBM SPSS Statistics for Windows version 27. Descriptive statistics (means, standard deviations, frequencies, and percent’s) were generated to summarize participant characteristics, knowledge domains, and confidence scores. For each public health domain, a composite knowledge score was calculated as the number of correct responses, and mean composite scores were compared between baseline and follow-up using paired sample t-tests. Mean differences in confidence were also examined across workshops. Missing data were minimum (<5%) and handled using pairwise deletion. Statistical significance was determined using *p* < 0.05.

During the final workshop (April 2022), qualitative data were collected FDGs lasting approximately 1 h each, conducted in either Lango or English according to participant preference. Each focus group included 9–10 participants resulting in three total focus groups (*N* = 29). Discussion followed a semi-structured guide with questions on the benefits and challenges of the YPHA program and recommendations for future activities. Sessions were audio recorded with participant consent, transcribed, and translated into English. USC GRIT students analyzed transcripts using DeDoose software to generate key themes on program impact and feasibility.

All evaluation activities received ethical approval by the USC Institutional Review Board, Gulu University Institutional Review Board, and the UNCST.

## Evaluation results

### Quantitative survey results

#### Baseline knowledge

At baseline, ambassadors demonstrated variable levels of public health knowledge across domains. The strongest areas included risky behaviors, water, sanitation and hygiene, and cancer and genetic disease awareness. The lowest baseline scores were observed in mental health, communicable disease prevention, and environmental health (Figure 3).

#### Gains in knowledge across workshops

Ambassadors exhibited statistically significant gains in overall public health knowledge following each of the first three training workshops (Figure 3). The largest improvements were observed in environmental health (24% increase) and non-communicable diseases (23% increase). By the conclusion of the third workshop, participants demonstrated the highest mastery in cancer and genetic diseases, injury prevention, nutrition and physical exercise, and COVID-19. Remaining knowledge gaps were identified in reproductive health, vector-borne diseases, and STI/HIV prevention and treatment.

Ambassadors’ understanding of COVID-19 prevention practices also remained consistently high throughout the program. Notably, vaccine hesitancy declined significantly as training progressed. Self-reported likelihood of getting the vaccine increased from 55 to 91% between workshops one and three.

#### Ambassador confidence and self-efficacy

Ambassadors reported substantial growth in confidence and self-efficacy related to public health communication (Figure 4). Confidence in communicating with diverse stakeholders rose markedly from baseline to Workshop 3, including a 54% increase when addressing large groups, 51% when engaging youth, 50% with adults, and 51% with local and national policymakers. Self-efficacy also improved considerably, with the proportion of ambassadors identifying themselves as “experts in disease prevention and control” increasing by 61% from baseline to Workshop 3.

### Focus group discussion results

Three major themes emerged from qualitative FDGs carried out at the conclusion of the fourth and final workshop: (1) personal growth and skill development; (2) program challenges and recommendations; and (3) and community applications and future aspirations.

### Personal growth and skill development

Ambassadors consistently described substantial personal growth, particularly in confidence, communication, and leadership. Several noted that participation in the YPHA program helped them overcome fears of public speaking and improved their ability to engage with diverse audiences. As one female ambassador explained, *“Last time I could not stand in front of people to talk in public, but now I talk to elders, children, and the community.”* Another participant emphasized the acquisition of practical knowledge, stating, “I *did not have any knowledge concerning health, but now I do.”* Several participants reflected on their emerging leadership abilities, with one male ambassador remarking, *“I did not know how to teach, now I can organize people, and they listen.”*

### Program challenges and suggestions for improvement

Although feedback was highly positive, ambassadors identified several challenges and offered constructive suggestions for future iterations of the program. Reported obstacles included environmental factors, such as adverse weather conditions, and occasional community resistance. Some ambassadors recalled, *“We faced challenges like insults from the community members, people started mocking us that we were doing free work.”* Others noted logistical difficulties, with one female participant stating, *“At times when it was raining, our books got wet, we need umbrellas for travel.”* Suggestions for improvement included expanding the program coverage to additional parishes and providing transportation support.

### Community applications and future aspirations

Ambassadors described numerous ways they applied their training within their communities, including constructing handwashing stations, distributing mosquito nets, and leading menstrual hygiene workshops. One female ambassador shared, *“The girls I taught are now making their own reusable pads - no more buying.”* Others observed tangible behavior changes in their communities: *“People now have latrines and rubbish pits - before they used to defecate in gardens.”* Nearly all participants expressed a desire to continue contributing to public health initiatives beyond the program. Several articulated aspirations for further education and professional advancement, such as one male ambassador who stated, *“My goal is to become a very good consultant. I am also planning to go back to school to become a doctor.”*

## Discussion

The peer-led YPHA program employed community-based participatory strategies to engage rural youth in public health research and practice during and after the COVID-19 pandemic. The program specifically aimed to overcome traditional barriers to youth engagement in decision making including diverse representation, access to digital technology, financial investment, and lack of trust and respect for youth perspectives among senior leaders. The YPHA model of participatory engagement offers a replicable framework for democratizing and decolonizing traditional top-down global health practice that have historically undervalued local knowledge and imposed extractive research methods.

The YPHA program successfully recruited and empowered marginalized rural youth often excluded from leadership initiatives. Consistent with literature emphasizing the potential of youth-focused CBPR projects in Africa, ambassadors both in and out of school demonstrated a nuanced understanding of the intersection between health and poverty, lack of opportunity, gender, and intergenerational power dynamics in their communities (12, 33–36). This aligns with broader CBPR theory, which highlights the effectiveness of inclusive research methods for effective capacity-building and community engagement (33, 37). They likewise contributed at all stages of public health research, including identifying community priorities, conducting data collection and analysis, and disseminating results (38). Ambassadors also engaged directly with local and national policymakers through press briefings, television interviews, and peer-reviewed publications, interpreting and disseminating findings and outputs in understandable and respectful ways that promoted community ownership (26). Their visibility and credibility were further enhanced through collaborations with partners such as the Peace Corps, which enlisted them to support malaria education and prevention campaigns. These activities helped overcome past CBPR program weaknesses in Africa and ensured strong community ownership of the project (39).

Evaluation findings underscore both individual and community benefits of this peer-to-peer approach. Similar to other youth-led participatory initiatives, the YPHA model fostered sustained engagement, critical thinking, problem-solving, communication, leadership, and teamwork (40, 41). The program also operated amid the extraordinary challenges of the COVID-19 pandemic. Uganda’s prolonged school closures lasting 83 weeks, the longest duration of school closures worldwide, disrupted education and curtailed international aid efforts, including the suspension of Peace Corps activities (42). As reported by ambassadors and corroborated by regional studies, these disruptions exacerbated social vulnerabilities and increased sexual violence, teenage pregnancy, and school dropout rates (43). The YPHA’s achievements must therefore be viewed within this constrained context, highlighting its reliance and adaptability.

The program also facilitated the development of social networks and capital at multiple levels, including within peer groups, bridging across communities and linking with institutional actors. These connections sustained youth engagement over several years and expanded access to educational and professional opportunities. Several ambassadors have since returned to school or pursued higher education. The program’s focus on social capital among peers also reduced past CBPR-related ethical challenges in sub-Saharan Africa, including those related to power imbalances between academic partners and community members (44, 45).

Outcomes from this program reflect those observed in evaluations of the benefits of CBPR, which suggests that participatory youth engagement in public health not only benefits individuals but also contributes to long-term societal development (46–48). Empowered youth are more likely to become civically active adults who contribute health equity and community resilience (49–51). The continued activity of YPHAs years after the program’s inception, supported by partners at USC, CCI, and EIA, demonstrates the sustainability of this model. Notably, the program achieved these outcomes with minimal financial investment; supported primarily by small community grants ranging from $3,000–$5,000 annually.

Several limitations warrant consideration. Despite careful translation and adaptation of materials with input from local officials and public health students at Makerere University, language nuances may have affected comprehension and response accuracy. Future iterations should further refine instruments to ensure linguistic and cultural appropriateness. Additionally, reliance on self-reported data and focus group feedback introduces potential social desirability bias. Nevertheless, the program’s robust mixed-methods design provides valuable insights for developing effective community-based youth health programs.

## Conclusion

Implementation of YPHA, a micro-interventional, community-based youth public health ambassador program, resulted in measurable improvements in knowledge, confidence, and engagement among rural Ugandan youth. By integrating CBPR principles, the program fostered youth leadership in promoting health behavior change, conducting local research, and addressing systemic barriers to health equity.

Given Sub-Saharan Africa’s high burden of disease and complex sociocultural landscape, youth-centered CBPR represents a particularly powerful mechanism for strengthening community resilience and advancing sustainable public health interventions. However, meaningful youth participation requires deliberate efforts to address community priorities, cultural norms, and structural constraints. The YPHA model provides a feasible, scalable framework for future youth-driven participatory health initiatives in the region. When integrated within broader health and social care systems, such community-anchored programs can accelerate behavior change, strengthen local capacity, and enhance population health and well-being, particularly among adolescents and young adults.

## Data Availability

The original contributions presented in the study are included in the article/supplementary material, further inquiries can be directed to the corresponding author.
